# Unleashing the efficacy of immune checkpoint inhibitors for advanced hepatocellular carcinoma: factors, strategies, and ongoing trials

**DOI:** 10.3389/fphar.2023.1261575

**Published:** 2023-08-31

**Authors:** Jiahui Yu, Mengnan Li, Boxu Ren, Le Cheng, Xiaoxiao Wang, Zhaowu Ma, Wei Peng Yong, Xiaoguang Chen, Lingzhi Wang, Boon Cher Goh

**Affiliations:** ^1^ School of Basic Medicine, Health Science Center, Yangtze University, Jingzhou, China; ^2^ Department of Haematology–Oncology, National University Cancer Institute, Singapore, Singapore; ^3^ NUS Center for Cancer Research (N2CR), Yong Loo Lin School of Medicine, National University of Singapore, Singapore; ^4^ Department of Pharmacology, Yong Loo Lin School of Medicine, National University of Singapore, Singapore, Singapore; ^5^ Cancer Science Institute of Singapore, National University of Singapore, Singapore, Singapore

**Keywords:** hepatocellular carcinoma, immune checkpoint inhibitors, combination therapy, preclinical study, clinical trial

## Abstract

Hepatocellular carcinoma (HCC) is a prevalent primary liver cancer, representing approximately 85% of cases. The diagnosis is often made in the middle and late stages, necessitating systemic treatment as the primary therapeutic option. Despite sorafenib being the established standard of care for advanced HCC in the past decade, the efficacy of systemic therapy remains unsatisfactory, highlighting the need for novel treatment modalities. Recent breakthroughs in immunotherapy have shown promise in HCC treatment, particularly with immune checkpoint inhibitors (ICIs). However, the response rate to ICIs is currently limited to approximately 15%–20% of HCC patients. Recently, ICIs demonstrated greater efficacy in “hot" tumors, highlighting the urgency to devise more effective approaches to transform “cold" tumors into “hot" tumors, thereby enhancing the therapeutic potential of ICIs. This review presented an updated summary of the factors influencing the effectiveness of immunotherapy in HCC treatment, identified potential combination therapies that may improve patient response rates to ICIs, and offered an overview of ongoing clinical trials focusing on ICI-based combination therapy.

## 1 Introduction

**FIGURE 1:**
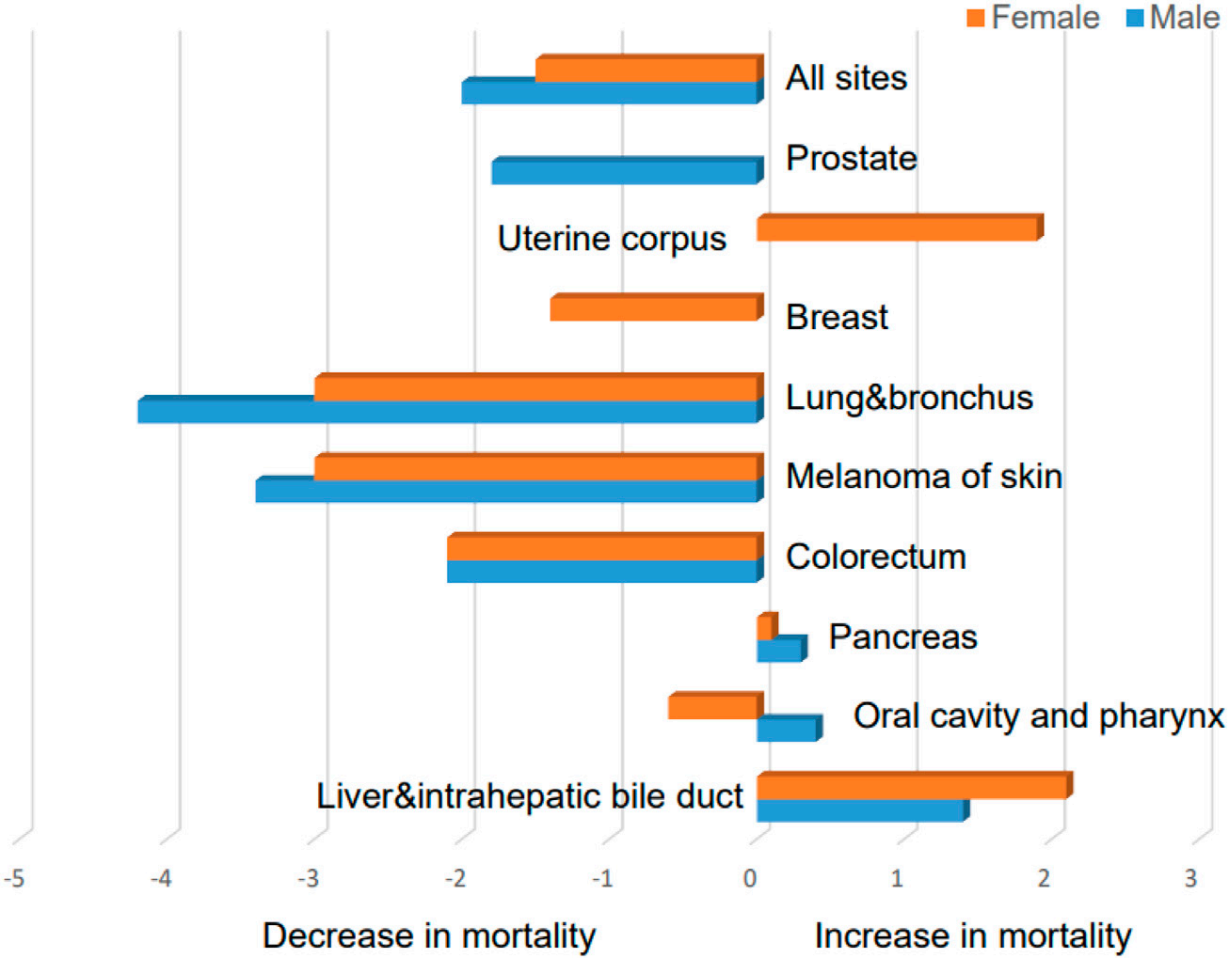
Average annual percent change for mortality rates with different malignancies in the United States between 2009 and 2018 (data were taken from Siegel, Rebecca L et al. “Cancer Statistics, 2021.” CA: a cancer journal for clinicians vol. 71,1 (2021): 7–33. doi:10.3322/caac.21654).

Globally, liver cancer remains a major health challenge due to an increasing incidence (Llovet et al., 2021). There are several types of liver cancers, but hepatocellular carcinoma (HCC) is the most common and contributes significantly to global cancer-related mortality as the sixth most frequently diagnosed cancer (Bray et al., 2018; Sung et al., 2021). During the period between 2009 and 2018, liver cancer has ranked first in terms of mortality increases in the United States (Figure 1). Various etiological factors, including chronic infections, such as hepatitis B virus (HBV) or hepatitis C virus (HCV), alcoholic and autoimmune hepatitis, nonalcoholic steatohepatitis (NASH), and metabolic diseases, make a contribution to the development of HCC, with variations observed between countries (Ozer Etik et al., 2017; Forner et al., 2018; Yang et al., 2019). Cirrhosis, regardless of its cause, poses a substantial risk for HCC (Marrero et al., 2018). Traditional treatment options for HCC include hepatectomy, liver transplantation, transarterial chemoembolization (TACE), selective internal radiation therapy, stereotactic body radiation therapy (SBRT), and ablation (European Association for the Study of the LiverEuropean Association for the Study of the Liver, 2018; Vogel et al., 2020; Vogel et al., 2021). Surgical resection is most effective for early-stage HCC. Unfortunately, the majority of the cases are diagnosed at advanced stages of the disease (Sperandio et al., 2022), limiting the feasibility of this treatment option and other local treatment, such as ablation and TACE, for a significant portion of patients (Zongyi and Xiaowu, 2020; Liu et al., 2021). Sorafenib and lenvatinib, along with cabozantinib, regorafenib, and ramucirumab as second-line options, have become the main therapeutic choices for advanced HCC patients (Gordan et al., 2020; Vogel et al., 2021; Zhang H. et al., 2022; Llovet et al., 2022). However, the treatment outcomes for advanced HCC remain unsatisfactory, necessitating further research to explore new therapeutic approaches.

The use of immunotherapy to treat solid tumors has emerged as a promising treatment option, including HCC, and has experienced rapid development in recent years. Immunotherapy enhances the body’s immune response, promotes tumor-specific immunity, and disrupts immune tolerance, thereby slowing down tumor progression (Fulgenzi et al., 2021; Sangro et al., 2021). ICIs play a key part in immunotherapy and were recognized with the 2018 Nobel Prize in Physiology or Medicine (Ballas, 2018). Major ICIs include PD-1, PD-L1, and CTLA-4 (Roudi et al., 2021). More than 10 types of cancer have shown efficacy with anti-PD-1/anti-PD-L1 monotherapy, including advanced melanoma, non-small-cell lung carcinoma (NSCLC), and renal cell carcinoma (Callahan et al., 2016; Hoos, 2016). The utilization of ICIs in the clinical management of advanced HCC has yielded notable therapeutic outcomes, leading to a transformative shift in the landscape of systemic HCC treatment strategies. ICI monotherapy, however, only produces 15%–20% objective responses in HCC (El-Khoueiry et al., 2017). Despite the strong antitumor properties of ICIs, a majority of HCC patients do not respond adequately. This may be due to the fact that HCC are usually cold tumors that do not respond well to ICIs. Therefore, there is an urgent need for some combination therapy strategies to convert HCC “cold" tumors into “hot" tumors, which are critical to improve the efficacy of ICIs in the treatment of advanced HCC. The present review delineates potential strategies aimed at transforming “cold" tumors into “hot" tumors, with the ultimate goal of ameliorating patient response rates to ICIs in the context of HCC. The review underscores the pivotal role of combination therapies and offers a comprehensive overview of ongoing preclinical and clinical trials centered on combination therapies involving ICIs. These concerted efforts are geared toward optimizing the therapeutic efficacy of ICIs in the management of HCC.

## 2 Literature search strategy

**FIGURE 2:**
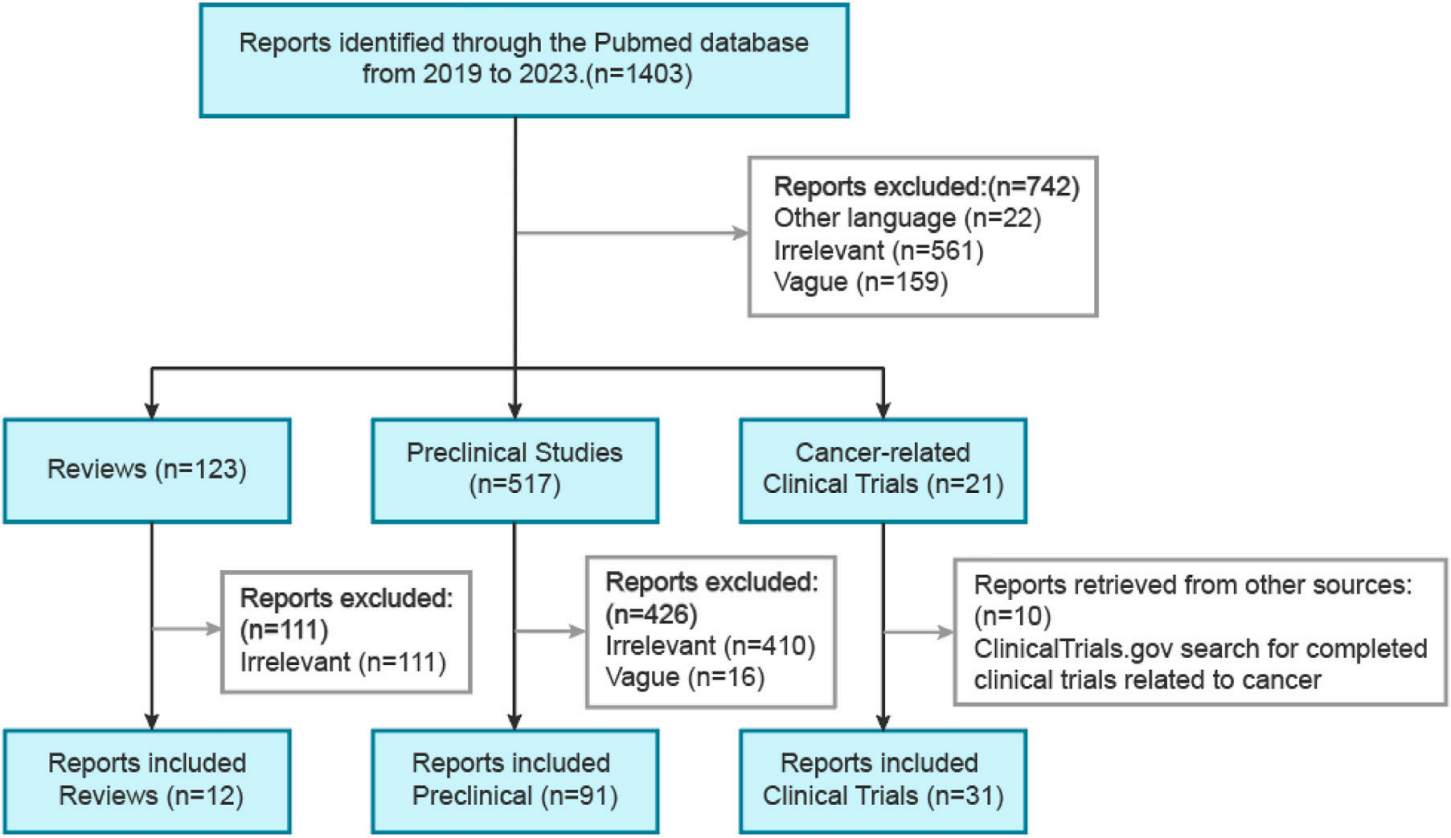
Primary search strategies for preclinical and clinical studies involving ICI combination therapy for HCC.

Through the use of the PubMed database, a systematic literature review was conducted, focusing on publications from the past 5 years. The initial search employed the keywords “hepatocellular carcinoma (topic) and immune checkpoint inhibitors (topic)," resulting in a total of over 1,200 publications. To refine the search and narrow down the results, a subsequent search was conducted employing the keywords “hepatocellular carcinoma (topic) and immune checkpoint inhibitors (topic) and combination therapy (topic)," which generated a total of 472 publications (Figure 2). Duplicate screening was carried out using EndNote (Endnote X9), and those studies that did not meet the predefined inclusion criteria were excluded based on a thorough evaluation of their titles and abstracts. The remaining studies were then categorized into reviews, preclinical studies, and clinical trials. Additional searches were conducted as necessary to retrieve further relevant information.

## 3 ICI targets and their immune inhibition

Cancer is a complex disease that elicits structural and functional changes in various human systems, including the immune system. In contrast, immunotherapy aims to restore antitumor immunity by enhancing the autoimmune defenses of the host immune system or eliciting novel immune reactions (Hiam-Galvez et al., 2021). Major types of immunotherapy include ICIs, pericyte therapy, therapeutic cancer vaccines, lytic virus therapy, and cytokine therapy (Zhang and Zhang, 2020). Cancer management has been transformed by immunotherapy in the past decade, with ICIs producing durable clinical responses and representing a promising immunotherapeutic approach. By obstructing suppressive immune checkpoints, such as CTLA-4, PD-1, and PD-L1, this therapeutic strategy strives to augment T-cell-mediated immunity. This is because T-lymphocyte activation and function are controlled by immune checkpoint molecules, which are inhibitory regulators of the immune system. Upon stimulation of the immune system, these entities are activated and serve as a regulatory mechanism, diligently preserving self-tolerance, mitigating autoimmune responses, and effectively governing the activation of the immune system (Syn et al., 2017). However, cancer cells, including those in the liver, exploit these molecules to avoid immune surveillance by activating immune checkpoints and suppressing T-cell activation (Chen and Flies, 2013). Prominent instances of suppressive immune checkpoint receptors encompass CTLA-4, PD-1, lymphocyte activation gene 3, and T-cell immunoglobulin and mucin domain-containing protein 3 (He and Xu, 2020).

CTLA-4, or CD152, is a suppressor receptor belonging to the CD28 immunoglobulin subfamily. A majority of its expression is seen on activated T cells but also available on Treg cells. The ligands of CTLA-4, namely, CD80, and CD86, are normally present on the surface of antigen-presenting cells (APCs) (Rowshanravan et al., 2018). Dual-signal stimulation is a necessary component for activating T cells while having an immune response. The T-cell receptor (TCR) recognition of the MHC/antigen peptide complex initiates an initial signal, conveying an antigen-specific recognition signal. The second signal relies on co-stimulatory molecules, mainly the interaction between CD28 receptors on T-cell and B7 (CD80/CD86) ligands on specialized APCs (Sharpe, 2009; Chen and Flies, 2013). Upon T-cell activation, CTLA-4 is expressed and binds to CD80 and CD86 in competition with CD28 ligands with higher affinity, thereby blocking CD28-mediated co-stimulation (van der Merwe and Davis, 2003; Teft et al., 2006). CTLA-4 and CD80 interact most strongly, while the affinity between CD28 and CD86 is the weakest (Rowshanravan et al., 2018). However, competition alone for ligand binding is not enough to completely eliminate co-stimulatory signals in T cells. CTLA-4 can also remove CD80 and CD86 ligands from APCs through transendocytosis and cytokinesis, effectively depriving APCs of their activation potential (Qureshi et al., 2011; Walker and Sansom, 2015).

There is still some debate regarding whether CTLA-4 provides inhibitory signals within the cell. It has been shown in some studies CTLA-4 disrupts the formation of zeta-associated protein of 70 kD (Zap70) microclusters (Schneider et al., 2008), while others propose different mechanisms (Calvo et al., 1997; Schneider et al., 2001). Similarly, some reports indicate that CTLA-4 alters the phosphorylation of the CD3ζ chain (Lee et al., 1998), while others present contrasting findings (Calvo et al., 1997). There has been a report that CTLA-4’s cytoplasmic tail recruits phosphoinositide 3-kinase (PI3K) (Hu et al., 2001), although other studies contradict this observation (Stein et al., 1994). Therefore, the precise mechanism of CTLA-4’s inhibition of T cells is not fully understood and requires further investigation. However, there is a consensus that CTLA-4 mainly inhibits T cells by competing with CD28 for ligand binding and by transendocytosing CD80 and CD86 to eliminate co-stimulatory signals. PD-1, also referred to as CD279, belongs to the CTLA4/CD28 family and is predominantly expressed in activated T lymphocytes, natural killer cells (NK cells), macrophages, dendritic cells (DCs), B cells, and monocytes (Guzik et al., 2019; Wu et al., 2022). PD-1 is a type I transmembrane protein with an extracellular IgV structural domain, an intermediate transmembrane region, and a cytoplasmic tail consisting of a total of 288 amino acids. PD-1 ligands comprise PD-L1 (also known as B7-H1 or CD274) and PD-L2 (also known as B7-DC or CD273). PD-L1 is broadly expressed in cancer cells, T cells, dendritic cells (DCs), B cells, and macrophages, and it assumes a prominent role in tumor immunity (Chen, 2004; Keir et al., 2008).

**FIGURE 3:**
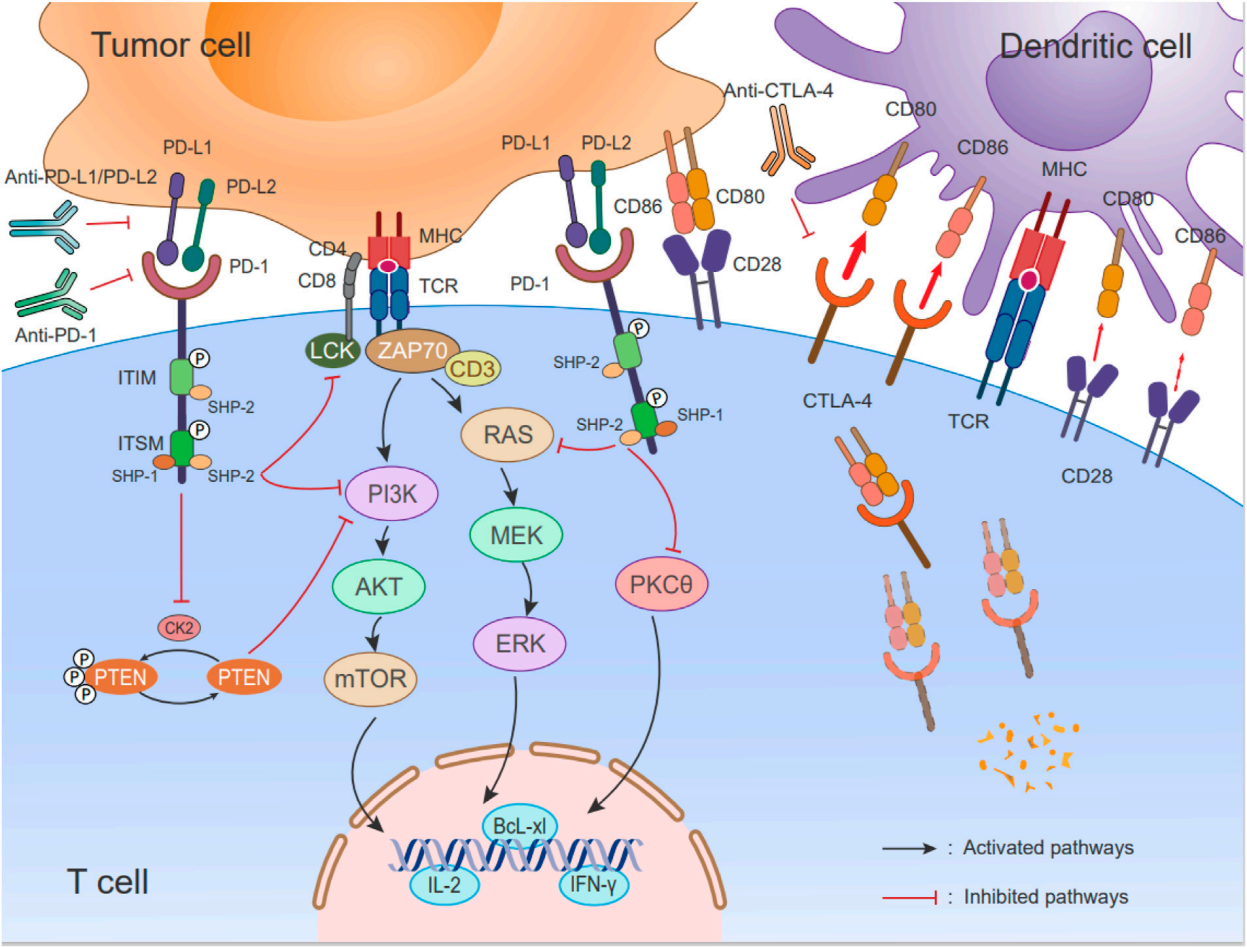
Immune checkpoints PD-1/PD-L1 signaling pathways and schematic of CTLA-4 cell biology. (Left) During antigen specificity, TCR interacts with the peptide/MHC complex and CD28 binding of B7-1 (CD80)/B7-2 (CD86) on APCs for co-stimulation. The expression of PD-1 increases upon activation of T cells, and it interacts with PD-L1/L2. When PD-1/PD-L1 signaling is activated, the phosphatase SHP-2 is recruited to the ITSM’s C-terminus, which inhibits the RAS-MEK-ERK and PI3K-Akt-mTOR pathways as well as LCK-induced ZAP70 phosphorylation. Additionally, there is a negative regulation of CK2 by PD-1, which phosphorylates PTEN, a negative regulator of the PI3K-Akt pathway. PTEN is inhibited by inhibiting CK2 protein function; PD-1 signaling reduces the activity of the PI3K pathway and decreases the expression of survival factor Bcl-xL. According to a recent report, PD-1 inhibits the activation of PKCθ, As a result, T cells secrete less cytokine, such as IFN-γ and IL-2. (Right) CD28 and CTLA-4 on the T cell bind to two ligands, CD80 and CD86, on DCs. The interactions occur at varying affinities (represented by the thickness of the arrows). CTLA-4 expressed in T cells is highly endocytic. This endocytosis removes its ligand (CD80 and CD86) that is a form of cell extrinsic competition.

Upon PD-1 binding to its ligand, Src homologous phosphatase 1 (SHP-1) and Src homologous phosphatase 2 (SHP-2) are recruited to the cytoplasmic tails of PD-1, which contain immunoreceptor tyrosine-based inhibitory motifs (ITIMs) and immunoreceptor tyrosine-based switch motifs (ITSMs). The ITIM recruits SHP-2, while the ITSM recruits both SHP-1 and SHP-2 (Sheppard et al., 2004). However, a higher affinity exists for the ITSM preferentially recruiting SHP-2, which inhibits downstream signaling of the TCR through signaling cascades (Yokosuka et al., 2012; Kuchroo et al., 2021). This leads to dephosphorylation of CD3ζ, Zap70, and protein kinase C θ (PKC-θ) (Sheppard et al., 2004). PD-1 also activates downstream Akt and inhibits PI3K activity by recruiting SHP2. The following should be noted: unlike PD-1, CTLA-4 only inhibits Akt activation and not PI3K activation (Parry et al., 2005). The PD-1 signaling pathway exerts its suppressor function in T cells by modulating the PI3K/Akt and Ras/MEK/Erk pathways. Specifically, PD-1 inhibits the phosphorylation of PTEN, which serves as a negative regulator of the PI3K-Akt pathway, achieved through the negative regulation of the CK2 protein (Wartewig et al., 2017). Inhibition of the PI3K-Akt pathway leads to reduced expression of the cell survival gene Bcl-XL, increased apoptosis of T cells, and decreased cytokine secretion. Another major signaling pathway targeted by PD-1 is the Ras/MEK/Erk pathway. PD-1 blocks the activation of this pathway, resulting in the inhibition of T-cell proliferation (Bivona et al., 2003; Patsoukis et al., 2012). Furthermore, PD-1 signaling attenuates the PKCθ/NF-κB signaling pathway, leading to a reduction in the levels of cytokines such as IFN-γ and interleukin-2 (IL-2) secreted by T cells (Sheppard et al., 2004; Wartewig et al., 2017) (Figure 3).

## 4 Immunophenotypic differences between “hot” and “cold” tumors

### 4.1 Updates on immunophenotype classification

The response to ICI therapy in cancer treatment varies among patients, and a subset of tumors that do not respond to this treatment are often referred to as “cold" tumors. There is little or no T-lymphocyte infiltration within the tumor parenchyma in these tumors. In contrast, “hot" tumors have a characteristic of a rich infiltration of T lymphocytes within the tumor parenchyma and tend to be more sensitive to ICI therapy (Chen and Mellman, 2017; Duan et al., 2020; Zhang J. et al., 2022).

The concept of hot and cold tumors was first described by Camus et al. in 2009. They analyzed the immune response in human colorectal cancer and identified three main features of immune coordination: hot, altered, and cold. The two-year risk of recurrence for these tumor types was found to be 10%, 50%, and 80%, respectively (Camus et al., 2009).

**FIGURE 4:**
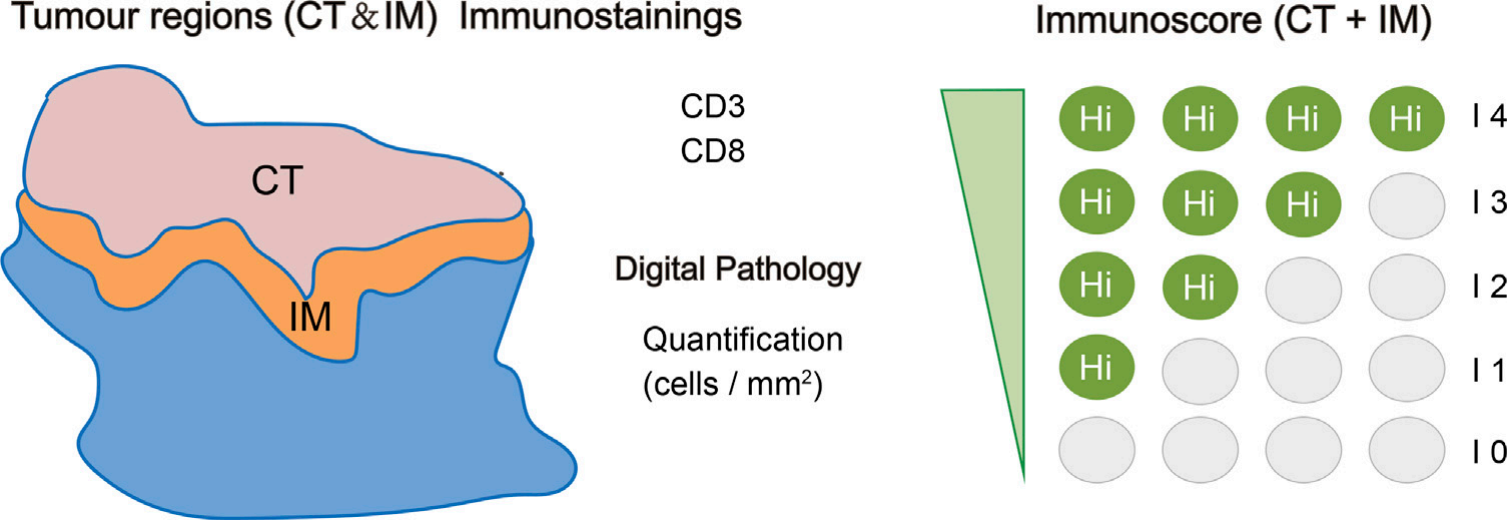
Specific immunoscore definitions and methods. The immunoscore is determined based on the quantification of two distinct lymphocyte populations (CD3/CD45RO, CD3/CD8, or CD8/CD45RO) present in both the core of the tumor (CT) and the invasive margin (IM) of tumors. This scoring system provides a range of scores, starting from immunoscore 0 (I0) indicating low densities of both cell types in both regions, up to immunoscore 4 (I4) representing high densities of these lymphocytes in both regions. The immunoscore, thus, serves as an essential metric for assessing the immune status within the tumor microenvironment, helping to better understand and predict the patient’s response to immunotherapeutic interventions (Galon et al., 2014).

Based on this study, the immunoscore, which is recommended by ESMO guidelines, proposed a classification system for tumors into five grades (Angell and Galon, 2013; Galon et al., 2014). This classification is based on quantitative measurements of CD3^+^ and CD8^+^ lymphocytes in both the tumor’s center as well as its border regions (Galon et al., 2006; Galon et al., 2013). CD3^+^ and CD8^+^ lymphocytes are absent from the center and border of tumors with an immune score of 0, indicating an immune desert phenotype. Tumors with an immune score of 4 have a high level of CD3^+^ and CD8^+^ lymphocytes in both regions, representing an immune-inflamed phenotype (Figure 4). The immune-altered phenotype is further categorized as two patterns: excluded and immunosuppressed. In altered-excluded tumors, lymphocytes are abundant at tumor margins but cannot penetrate into the tumor core. In altered-immunosuppressed tumors, lymphocytes are present in both the central and marginal regions, but the cell density is low (Galon and Bruni, 2019).

Immunoinflammatory tumors, often referred to as “hot" tumors, exhibit distinctive features such as heightened levels of T-cell infiltration, PD-L1 expression, tumor mutation burden (TMB), and an abundance of pro-inflammatory cytokines. (Powles et al., 2014; Taube et al., 2014). These tumors indicate the existence of a pre-existing anti-tumor immune response, which, in some cases, might be impeded or suppressed by immune checkpoint inhibitors (ICIs) targeting molecules such as PD-1/PD-L1. Patients with immunoinflammatory tumors tend to show stronger responses to anti-PD-1/PD-L1 therapy in clinical settings, although not all patients with these tumors exhibit this response. Tumor-infiltrating immune cells (TILs) are necessary but not sufficient for generating a response (Chen and Mellman, 2017).

On the other hand, “cold" tumors, which exhibit immune desert and immune rejection features, have the following characteristics: low PD-L1 expression, low mutational burden, diminished expression of antigen presentation markers, and poor T-cell infiltration, leading to immune ignorance (Hegde et al., 2016). These tumors also contain soluble suppressive mediators (e.g., TGFβ, IL-10, and VEGF) and immunosuppressive cells (e.g., myeloid-derived suppressor cells and regulatory T cells) (Galon and Bruni, 2019). “Cold" tumors are either immune deficient or have impaired innate immune functions due to T-lymphocyte rejection. Compared to “hot" tumors, “cold" tumors show limited response to ICI monotherapy (Herbst et al., 2014).

### 4.2 Biomarkers for ICI treatment

Alongside the classification of immunophenotypes, the utilization of biomarker-based stratification holds significant promise in predicting the efficacy of immunotherapy in HCC patients. By tailoring treatment to individuals who are more likely to benefit from immunotherapy, this approach seeks to maximize the effectiveness of ICIs and achieve better treatment outcomes for HCC patients. Within this context, we present a summary of the predictive biomarkers currently used to predict the likely response to ICIs in HCC patients, derived from both intratumoral and extratumoral tissues.

#### 4.2.1 Intra-tumoral tissue biomarkers

PD-L1 expression has been utilized as an initial biomarker for predicting the response to anti-PD-1 or anti-PD-L1 therapy. Generally, it has been observed that response rates are higher in patients classified as PD-L1 positive in comparison with those classified as PD-L1 negative. Notably, as a therapeutic biomarker, PD-L1 immunohistochemistry has been approved by the FDA for predicting treatment outcomes in advanced NSCLC and bladder cancer (Gibney et al., 2016). However, within the context of HCC, the use of PD-L1-based biomarkers has yielded conflicting results in clinical trials. Notably, in trials like KEYNOTE-224 and CheckMate 459, the treatment with nivolumab and pembrolizumab demonstrated better outcomes in patients with PD-L1-positive tumors as compared to those with PD-L1-negative tumors in HCC. Conversely, the CheckMate 040 and NCT02658019 trials reported that the response rate to immunotherapy was not significantly different based on PD-L1 expression levels in HCC patients. These contradictory findings may be attributed to limitations in detecting PD-L1 levels and the relatively small sample sizes employed in these studies.

TMB is a molecular biomarker that quantifies the number of nonsynonymous mutations in the genome of somatic cells within a tumor. It has been investigated as a predictive biomarker for ICI response across various tumor types. A crossover study involving multiple cancer types demonstrated that tumors with higher TMB exhibited better responses to PD-1/PD-L1 therapy, and there was a positive correlation between TMB and PD-L1 expression on tumor cells (Yarchoan et al., 2017). Additionally, a large-scale study involving a substantial number of patients with advanced cancer has confirmed that higher tumor mutation burdens (TMBs) are linked to improved survival outcomes with ICIs across a wide range of cancer types (Samstein et al., 2019). However, one study analyzing genomic biomarkers in 755 patients found that the median TMB was four mutations per megabase (Mb), and only a small percentage of HCC tumors (0.8%) had high TMB. Another small case study with 17 patients did not find a significant correlation between TMB and treatment efficacy (Ang et al., 2019). These discrepancies could be attributed to the absence of standardized thresholds for TMB assays and variations in quantification methods (Merino et al., 2020). Consequently, more research studies will be needed to fully understand the value of TMB as a predictor of ICI effectiveness in HCC.

Certain mutations or alterations in tumor-related genes, including Wnt/β-catenin pathway alterations and TP53 gene mutations, have been associated with resistance to ICIs and the development of an immunosuppressive tumor microenvironment (TME) in advanced HCC. Mutations activating the Wnt/β-catenin signaling pathway, particularly in the CTNNB1 gene, are characteristic of the immunoexclusion class (cold tumors) in HCC (Pinyol et al., 2019). Prospective next-generation sequencing studies by Harding et al. provided predictive and prognostic insights for HCC patients receiving systemic therapy, revealing frequent alterations in TP53 (33%) and the Wnt/β-catenin pathway (45%), which represent mutually exclusive molecular subsets (Harding et al., 2019). Among HCC patients treated with ICIs, those with activated Wnt/β-catenin pathway alterations showed reduced disease control rates (0% vs. 53%), shorter median progression-free survival (mPFS) (2.0 vs. 7.4 months), and shorter median overall survival (mOS) (9.1 vs. 15.2 months) in comparison with patients with wild-type Wnt HCC (Harding et al., 2019). There has also been a report of the activation of the catenin pathway contributing to promote immune evasion and PD-1 resistance in HCC, while the expression of chemokine (C-C motif) ligand 5 (CCL5) in MYC has shown potential in restoring immune recognition of HCC (Ruiz de Galarreta et al., 2019). The downregulation of peptidoglycan recognition protein 2, a liver-specific pattern-recognition receptor, in HCC has been involved in poor prognosis, while its overexpression in HCC cells enhanced anti-tumor immune responses in mice (Yang et al., 2020). TP53 mutations, the most common in HCC, have been closely linked to the immune microenvironment, with tumors harboring TP53 alterations exhibiting reduced infiltration of CD3^+^ T cells and increased infiltration of Foxp53+ Treg cells, leading to immune response downregulation (Hu ZQ. et al., 2021). TP53 dysfunction has also been involved in high chromosomal instability and features of immune rejection in HCC, although conflicting results have been reported, with some studies suggesting that TP53 mutations were involved with increased cytotoxic lymphocyte infiltration and longer overall survival (Bassaganyas et al., 2020).

The medical oncology community has extensively discussed gene alterations associated with DNA damage repair (DDR) as potential biomarkers for predicting response to ICIs. In HCC patients receiving ICI treatment, Chen et al. found that those with high expression of DDR-related genes experienced longer survival compared to those with low expression (Chen Y. et al., 2021). Interferon signaling and major histocompatibility complex-related genes have also emerged as important molecular features in HCC’s response to ICIs. By conducting a comprehensive analysis of genomic expression data, mutation profiles, and histological evaluations in a cohort comprising 111 HCC patients, researchers successfully developed an 11-gene signature which is able to predict response and survival outcomes in patients undergoing first-line anti-PD-1 therapy (Haber et al., 2023). Another study identified five immune-related genes (LDHA, BFSP1, PPAT, NR0B1, and PFKFB4) and constructed an innovative prognostic model on the basis of these genes, enabling the stratification of HCC patients into low-risk and high-risk groups. The low-risk group exhibited better prognosis and greater sensitivity to immunotherapy compared to the high-risk group (Gu et al., 2021). Cancer stem cells, known to promote the initiation of tumors, metastasis, and drug resistance in HCC, have also been investigated. A stemness-related classifier was developed using machine learning algorithms and RNA-seq data from CSC-associated HCC clusters. This classifier has potential applications in predicting response to immunotherapy in HCC patients (Chen D. et al., 2022). Additionally, studies have explored the role of macrophage-related genes and cancer-associated fibroblast (CAF)-associated genes as potential predictors of ICI response (Wang T. et al., 2022). Predictive models integrating gene expression profiles and biological phenotypes, such as tumor immune characteristics and mutation profiles, have been established. Furthermore, the expression levels of CDK1, CCNB1, and CCNB2 have been associated with immune cell infiltration, including CD4^+^ T cells, CD8^+^ T cells, and DCs, suggesting their potential as predictive biomarkers for HCC (Zou et al., 2020).

#### 4.2.2 Extra-tumoral tissue biomarkers

In recent years, extratumoral tissue biomarkers, commonly known as liquid biopsy, have gained significant attention as promising biomarkers in cancer research, including HCC. Liquid biopsies offer a non-invasive approach and have found extensive applications in the field, particularly in the context of ICI treatment. Compared to tissue biopsies, peripheral blood biomarkers can be easily collected and repeatedly assessed during ICI therapy, providing more convenience for clinical application.

Alpha-fetoprotein (AFP) has long been recognized as a widely used biomarker for HCC. Recent studies have shown that a decline in early serum AFP levels is significantly related to improved objective response and survival following ICI treatment in patients with advanced HCC (Lee PC. et al., 2020). Nevertheless, the CheckMate 040 trial indicated that patients with baseline AFP levels <400 μg/L had longer OS compared to those with AFP ≥400 μg/L, but the objective response rate (ORR) and the disease control rate (DCR) were similar irrespective of the baseline AFP level (Sangro et al., 2020).

Furthermore, the Scheiner’s group proposed the combined detection of serum AFP and C-reactive protein (CRP) to enhance the prognosis prediction of HCC patients treated with ICI. Their CRAFITY score, based on AFP and CRP levels, has been widely discussed in the academic community (Table 1). Retrospective training cohorts (190 cases) and validation cohorts (120 cases) confirmed that patients with CRAFITY scores of 0, 1, and 2 had median OS of 27.6 months, 11.3 months, and 6.4 months, respectively, after receiving immunotherapy (Yang et al., 2022). The radiological response was also best when patients have a CRAFITY score of 0 (Yang et al., 2022). This scoring system has shown predictive efficacy for immunotherapy in HCC patients and has been validated in both foreign and domestic populations, aiding clinicians in patient stratification and the development of personalized treatment plans.

**TABLE 1:** CRAFITY scoring rules.

CRAFITY score = AFP score + CRP score		1 point	AFP≥100 ng/ml	CRP≥1 mg/dl
		0 points	AFP<100 ng/ml	CRP<1 mg/dl
Total score	0 points	AFP<100 ng/ml and CRP<1 mg/dl		
	1 point	AFP≥100 ng/ml or CRP≥1 mg/dl		
	2 points	AFP≥100 ng/ml and CRP≥1 mg/dl		

Additionally, SLFNs (Schlafen proteins) have been investigated as potential predictive biomarkers for ICI treatment. A significant increase in SLFN11 was found in tumors that responded to ICI therapy. In HCC cells lacking SLFN11, macrophage migration and M2-like polarization are induced in a C-C motif chemokine ligand 2 (CCL2)-dependent manner, resulting in increased PD-L1 expression. Thus, patients with high serum SLFN11 levels are more likely to benefit from ICIs (Zhou et al., 2023). Moreover, plasma interleukin-6 (IL-6) has shown potential as a predictive biomarker in patients with advanced HCC receiving combination immunotherapy (Atezo/Bev) (Myojin et al., 2022).

These emerging extratumoral tissue biomarkers, including AFP, CRAFITY score, SLFNs, and plasma IL-6, hold promise for improving the prediction of treatment response in HCC patients undergoing immunotherapy. Their non-invasive nature and potential clinical utility make them valuable tools in personalized medicine approaches.

Liquid biopsy has become an increasingly valuable approach in cancer research over the last decade, and several components can be analyzed, including circulating tumor DNA (ctDNA), circulating tumor cells (CTCs), exosomes, and circulating RNA (such as microRNA) (Nikanjam et al., 2022). ctDNA refers to fragments of DNA originated from tumor cells that circulate in the blood, primarily originating from necrotic or apoptotic tumor cells and CTCs. ctDNA is promising for predicting response to ICIs. A prospective clinical trial demonstrated that cancer patients treated with anti-PD-L1 antibodies exhibited a notable association between ctDNA levels and tumor size. Furthermore, the ctDNA levels were identified as prognostic factors for treatment response rate, PFS, and OS (Cabel et al., 2017).

In peripheral blood, cell-free DNA (cfDNA) including ctDNA, reflects molecular abnormalities present in tumor tissue. Matsumae et al. investigated whether cfDNA/ctDNA could serve as predictive markers for treatment outcomes in patients with unresectable hepatocellular carcinoma (u-HCC) receiving anti-PD-L1/VEGF therapy (Atezo/Bev). The results of the study suggest that patients with high plasma cfDNA levels had lower response rates, PFS, and OS compared to those with low plasma cfDNA levels. Additionally, the presence of ctDNA was found to be associated with adverse prognostic factors, including higher neutrophil-to-lymphocyte ratio (NLR), larger tumor size, and the presence of microvascular invasion (MVI). Commonly mutated genes in HCC, such as TERT promoter, TP53, and CTNNB1, were detected through ctDNA analysis. The OS of HCC patients with TERT mutations was significantly shorter than that of patients without TERT mutations, although there was no significant difference in treatment response and PFS to Atezo/Bev between the two groups, warranting further validation (Matsumae et al., 2022). However, the utility of ctDNA as an HCC biomarker has limitations, including low levels for early detection and non-standardized procedures for preparing samples and analyzing data.

CTCs expressing PD-L1 can also be used as biomarkers for predicting treatment effects in patients with HCC and undergoing ICI immunotherapy. Winograd et al. analyzed HCC CTCs expressing PD-L1 and found that PD-L1-positive CTCs were predominantly present in advanced HCCs and were independent predictors of OS. In HCC patients receiving anti-PD-1 therapy, the presence of PD-L1+ CTCs was strongly correlated with a favorable treatment response (Winograd et al., 2020). However, CTCs have limitations as an HCC biomarker, including difficulties in accurate early detection and their usually low frequency, which may require combined detection approaches to increase detection rates.

### 4.3 Tendency towards the immunologically “cold” state in the development of HCC

The liver has a unique immune microenvironment characterized by its role in metabolism and exposure to microbial products. While the liver needs to establish immune tolerance to harmless foreign molecules, it also requires an effective immune response to pathogens (Gao et al., 2008). Various resident cells in the liver, such as liver sinusoidal endothelial cells, Kupffer cells, hepatocytes, and DCs, functionally suppress the adaptive immune response under normal conditions, maintaining immune non-responsiveness. However, when the liver is invaded by viruses like HBV or HCV, the balance between tolerance and immunity is disrupted, resulting in an ineffective or transient initiation of cytotoxic T lymphocytes. These immunosuppressive responses are crucial for liver homeostasis but can also contribute to immune evasion in HCC (Feng et al., 2021).

Due to its unique immune microenvironment, HCC often presents as an immunologically “cold" tumor with a low response rate to ICIs in comparison with other cancers, such as melanoma and NSCLC. The response rate to ICIs in HCC is approximately 15%–20%. The immuno-hyporesponsiveness of the liver contributes to the limited efficacy of immunotherapy in HCC.

In summary, the liver’s specific immune microenvironment, characterized by immune tolerance and suppression, contributes to the immunologically “cold" nature of HCC and its limited response to ICIs. Understanding and overcoming the immune evasion mechanisms in the liver are crucial for improving immunotherapeutic strategies in HCC.

## 5 Strategies for converting “cold” tumors into “hot” tumors

**FIGURE 5:**
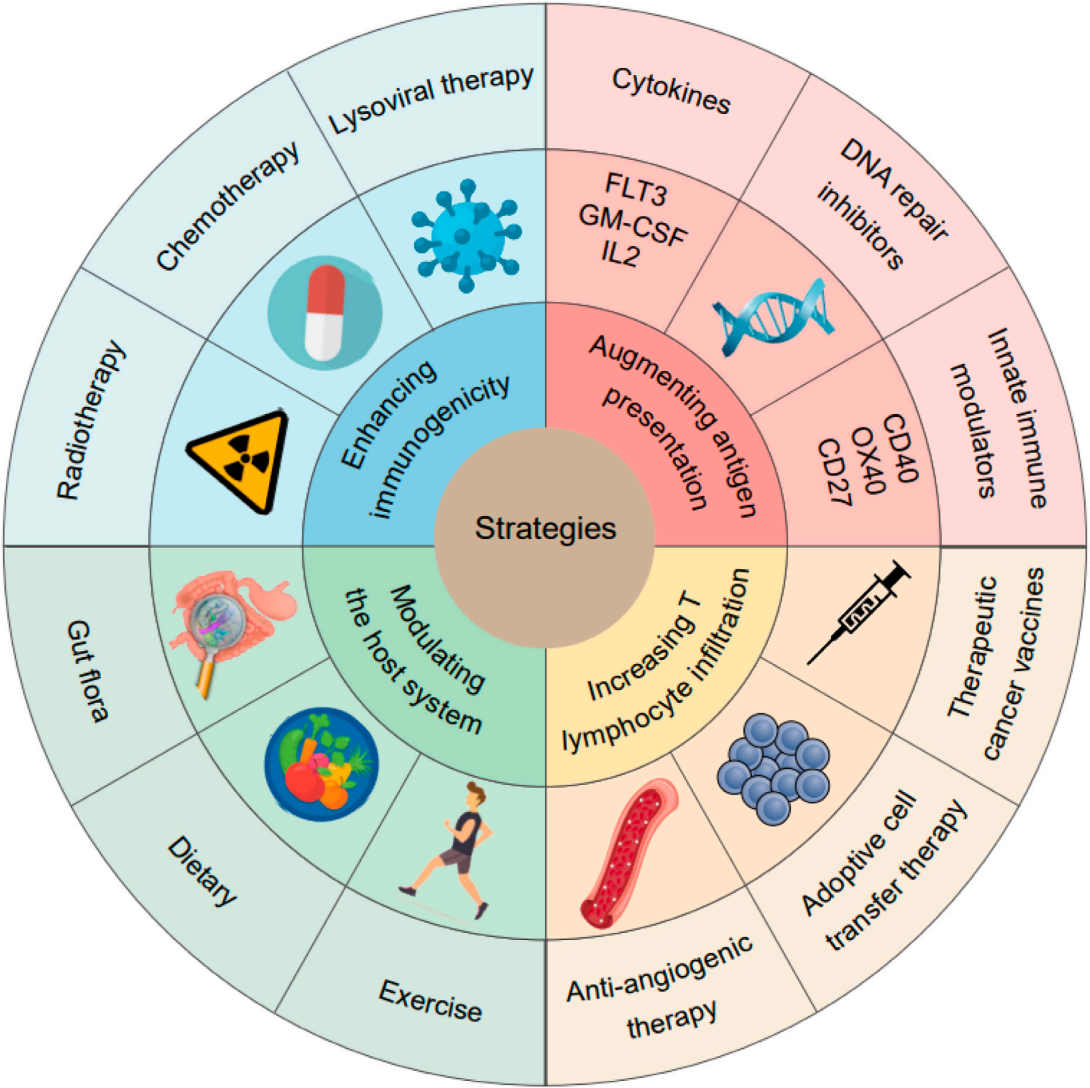
Strategies for converting “cold” tumors into “hot” tumors.

To enhance the response of HCC to ICIs, strategies can be employed to modify the TME from a “cold" state to a “hot" state. These approaches aim to increase the immunogenicity of HCC tumor cells, improve antigen presentation, increase the recruitment and infiltration of effector T lymphocytes, and modulate the host system (Figure 5). Some of these strategies include the following.

First, enhancing immunogenicity: various interventions such as radiotherapy, chemotherapy, and lysoviral therapy can be used to enhance the immunogenicity of HCC tumor cells. In addition to inducing immunogenic cell death, these treatments can release antigens associated with tumors and promote the release of danger signals that activate the immune system.

Second, augmenting antigen presentation: agents such as cytokines, DNA repair inhibitors, and innate immune modulators can be utilized to enhance antigen presentation. Cytokines can promote the expression of major histocompatibility complex (MHC) molecules on tumor cells, facilitating recognition by T cells. DNA repair inhibitors can increase the accumulation of DNA damage, leading to the release of antigens specific to tumors. Innate immune modulators can activate APCs, promoting efficient antigen presentation.

Third, increasing T-lymphocyte infiltration: therapies aimed at increasing the recruitment and infiltration of effector T lymphocytes into the TME can improve the response to ICIs. Tumor vasculature can be normalized with anti-angiogenic therapies, allowing better T-cell infiltration. Adoptive cell transfer therapy involves the infusion of tumor-specific T cells into patients to enhance antitumor immunity. Therapeutic cancer vaccines can also improve the immune system’s function to generate tumor-specific T lymphocytes.

Last, modulating the host system: modifying the host system can impact the TME and immune response. Regulating the gut flora through approaches such as probiotics or fecal microbiota transplantation can influence systemic immune function. Dietary interventions and exercise can also affect the immune system and potentially enhance the response to ICIs.

These strategies aim to overcome the immunosuppressive characteristics of the HCC microenvironment, promote antitumor immune responses, and improve the efficacy of ICIs in HCC treatment. It will be necessary to conduct further research and clinical studies in order to validate and optimize these approaches.

### 5.1 Improving the immunogenicity of HCC

The level of tumor immunogenicity is a pivotal factor that significantly influences a patient’s response to ICIs. Tumors characterized by high immunogenicity hold a theoretical advantage in terms of responsiveness to immunotherapy, in contrast to tumors with low or negligible immunogenicity. This crucial attribute is shaped by the intricate interplay of the TME and various associated factors, while also being inherently governed by the tumor cells themselves. The immunogenicity of tumor cells is determined by two distinct factors: tumor antigenicity and the efficiency of antigen processing and presentation (Blankenstein et al., 2012). Several studies have shown that impaired antigen presentation leads to treatment failure with ICIs (Chowell et al., 2018). Moreover, tumor cell immunogenicity not only impacts the local tumor environment but also influences the likelihood of tumor recurrence and metastasis. Therefore, strategies aimed at augmenting tumor immunogenicity have the potential to enhance response rates to immunotherapy by converting immunotherapy non-responsive tumors into immunotherapy-responsive entities.

#### 5.1.1 Radiation therapy

Radiotherapy has shown promise as a therapeutic approach in combination with ICIs due to its immunostimulatory properties. By inducing immunogenic cell death (ICD) in tumor cells, radiotherapy can elicit an immune response accompanied by the release of specific signaling molecules assumed to be damage-associated molecular patterns (DAMPs), including high mobility group protein 1 (HMGB1), calreticulin (CRT), heat shock proteins (HSP70, HSP90), and adenosine-5′-triphosphate (ATP) (Rodriguez-Ruiz et al., 2019). HMGB1 binds to chromatin and activates DCs through Toll-like receptor 4 (TLR4) and late glycosylation end-product receptors, leading to T-lymphocyte activation (Kawai and Akira, 2010). Similarly, CRT, a calcium-binding protein of the endoplasmic reticulum, promotes DC maturation and pro-inflammatory cytokine production in APCs, such as IL-6 and TNFα (Obeid et al., 2007). ATP, an intercellular signaling factor in the extracellular matrix, triggers the activation of DC inflammatory vesicles by binding to the P2X7 purinergic receptor, resulting in the secretion of IL-1β and subsequent induction of interferon-γ (IFNγ) production, facilitating the generation of CD8 T cells (Golden et al., 2014). There is a phenomenon called the “distant effect" observed in clinical cases of HCC, where ICD induced an antitumor immune response leading to the regression of distant tumors (Okuma et al., 2011). Thus, enhanced ICD signaling, including HMGB1 release and calreticulin expression, represents a mechanism by which radiation therapy directly initiates immune responses and synergizes with immunotherapy.

Radiation therapy elicits immune activation through various mechanisms, including the upregulation of major histocompatibility complex class I (MHC-I) expression, which facilitates antigen presentation to CD8^+^ T cells. In HCC, the downregulation of MHC-I expression contributes to immune evasion, whereas radiation enhances MHC-I molecules and antigen presentation, crucial for immune recognition (Reits et al., 2006). Moreover, IFNs play a significant role in radiation-induced immune activation. Type I IFNs are upregulated following irradiation through the cyclic GMP-AMP (cGAMP) synthase (cGAS)-stimulator of interferon genes (STING) pathway in DCs (Du et al., 2022). The cGAS enzyme detects cytoplasmic DNA and produces the second messenger cGAMP, which activates the adaptor protein STING. Radiation-induced type I IFNs enhance DC cross priming, induce tumor regression, and augment radiation-induced antitumor immune responses (Deng et al., 2014a). Furthermore, preclinical studies have demonstrated that radiation can increase immune checkpoint ligands expression, including PD-L1, in the TME. The upregulation of PD-L1 mediates immunosuppression, which can be relieved by anti-PD-1/PD-L1 antibodies, highlighting the importance of combination therapy (Deng et al., 2014b; Dovedi et al., 2014). Collectively, these findings support the potential of radiation therapy as a valuable tool combining ICIs, as it enhances tumor susceptibility to immune responses.

#### 5.1.2 Chemotherapy

Despite its historical reputation as an immunosuppressive agent that potentially reduces immune cells crucial for antitumor immunity, chemotherapy has shown positive in multiple clinical trials as an approach to increase the immunogenicity of HCC tumor cells. Chemotherapy, by inducing DNA damage, can trigger ICD, creating an inflammatory TME and promoting the production of neoantigens that stimulate immune response that is effective against tumors (Ochoa de Olza et al., 2020). This mechanism involves the activation of type I interferon signaling in tumor cells and increased recruitment of APCs to the TME (Brown et al., 2018). Moreover, platinum-based chemotherapy upregulates the expression of MHC-I and reverses tumor immune evasion. Studies have shown that cisplatin promotes the infiltration of inflammatory APCs into tumors, which express higher levels of CD70, CD80, and CD86 co-stimulatory ligands, leading to the induction and activation of tumor-specific CD8^+^ T cells (Beyranvand Nejad et al., 2016). The elevation of inflammatory cytokines can also activate the angiogenic network, converting a non-immunogenic microenvironment into an immunogenic one and attracting cytotoxic T cells to the tumor (Huang et al., 2021). Furthermore, chemotherapy enhances the activity of tumor-killing immune cells (CD4^+^ and CD8^+^ T cells) in the TME while reducing the activity of suppressor immune cells (regulatory T cells and myeloid-derived suppressor cells) (Xue et al., 2021). Over the years, several chemotherapeutic agents such as doxorubicin, paclitaxel, oxaliplatin, gemcitabine, bortezomib, and mitoxantrone have demonstrated the ability to induce ICD and immunogenicity in clinical settings through diverse mechanisms. For instance, recent studies have highlighted the pro-inflammatory response induced by very low doses of paclitaxel in different cancer cells, dependent on circulating cGAS and STING, leading to IFN signaling activation and enhancement of tumor immunogenicity (Serpico et al., 2023). Chemotherapy can be administered directly through interventional transarterial routes in addition to systemic administration. TACE or drug-eluting bead TACE (DEB-TACE), widely used in HCC treatment, is the standard of care for intermediate-stage (BCLC-B) HCC patients (Gordan et al., 2020). TACE blocks tumor blood supply by injecting embolic agents into the supplying vessels at the lesion site via intra-arterial catheters, delivering high concentrations of chemotherapeutic drugs directly to the tumor for maximal cytotoxic effect. Adriamycin, a commonly used chemotherapy agent in TACE, is considered immunogenic, enhancing ICD and immune activation (Apetoh et al., 2008). In one study, changes in peripheral immune cell subsets before and after TACE treatment in HCC patients were investigated. The findings revealed an increased ratio of CD4+/CD8+ T lymphocytes, higher frequency of Th17 cells, and a decrease in Treg cells, significantly improving immune function in patients with HCC (Liao et al., 2013; Liao et al., 2015). In conclusion, combining chemotherapy with ICIs can enhance the immune response against tumors.

#### 5.1.3 Oncolytic viruses

Oncolytic viruses (OVs) belong to a distinct class of agents that possess innate immune-stimulating properties, effectively activating immune responses, both innate and adaptive. Cancer cells are selectively targeted and eliminated by these viruses, sparing normal cells, which makes them a promising therapeutic option (Chiocca and Rabkin, 2014). OVs exert their effects on the cancer immune cycle by selectively replicating within-tumor cells, inducing ICD, and releasing danger signals and soluble antigens. This process recruits immature DCs and innate lymphoid-like cells, initiating a nonspecific immune response and correcting antigen processing and presentation defects (Bommareddy et al., 2018). Moreover, OVs can induce a pro-inflammatory immune response in the TME, leading to antitumor and antiviral effects. They have the ability to convert “immunocold" tumors into “immunohot" tumors, promoting immune cell infiltration and enhancing cytokine activity (Ylosmaki and Cerullo, 2020). JX-594, the most extensively studied OV for HCC, has entered clinical trials. Research has shown that JX-594 not only directly lyses cancer cells but also stimulates the immune response against cancer by increasing tumor antigens and expressing modified hGM-CSF to enhance immune responses (Park et al., 2008; Heo et al., 2011; Heo et al., 2013; Yoo et al., 2017). Nevertheless, further investigation is required to determine the ideal intra-tumor immune activator and the most suitable lysovirus for specific immunotherapeutic strategies.

### 5.2 Enhanced antigen presentation

Antigen presentation has a crucial impact on bridging innate and adaptive immunity, with APCs such as DCs, monocytes-phagocytes, and B cells serving as key players. Among these, DCs are specialized APCs known for their competence to activate CD4^+^ or CD8^+^ T cells. DCs encompass different subsets, including conventional CD11c+ dendritic cells (cDCs), plasmacytoid dendritic cells (pDCs), and cross-presenting CD8α+ or CD103+ DCs. Studies have highlighted the importance of enhancing antigen presentation to enhance the efficacy of ICIs.

#### 5.2.1 Cytokines

FMS-like tyrosine kinase 3 (FLT3) is a receptor tyrosine kinase of the type III category. Its ligand, FLT3L, acts as a growth factor for DCs, regulating their development in the bone marrow and lymphoid organs, as well as promoting DC proliferation at tumor sites (Cueto and Sancho, 2021; Lutz et al., 2022). Studies have confirmed the potential of FLT3L in HCC treatment, including *in situ* vaccination approaches using recombinant adenovirus expressing FLT3L (Kawashita et al., 2014a). These studies have shown that Adeno-FLT3L can stimulate endogenous DC proliferation, enhance the effectiveness of radiation therapy combined with gene therapy, and induce a Th1-polarized immune response, leading to improved antitumor responses (Kawashita et al., 2014a). FLT3L has also been associated with liver fibrosis regression and expansion of conventional and plasmacytoid DC populations in peripheral lymphoid organs. Granulocyte-macrophage colony stimulating factor (GM-CSF) is a cytokine that promotes the differentiation and proliferation of macrophages and DCs. Among its uses are attracting and stimulating intra-tumoral DCs in various solid tumors (Francisco-Cruz et al., 2014). The use of GM-CSF-expressing poxvirus JX-594 in HCC has shown antitumor activity (Heo et al., 2013). However, GM-CSF has also been linked to HCC carcinogenesis and has immunosuppressive effects in the TME (Lin et al., 2017). GM-CSF can be combined with IL-2 to enhance the efficacy of IL-2 agonists, driving DC differentiation and proliferation, and recruiting activated CD8^+^ T cells, NKT cells, and macrophages (Chang et al., 2007). IL-2 has been used as an immunotherapy for cancer treatment, particularly in advanced renal cell carcinoma and melanoma (Dimitrijevic et al., 2012). Although high-dose IL-2 administration can lead to durable responses, it carries significant risks of toxicity. Low-dose continuous infusion IL-2 therapy has been developed as an alternative with demonstrated efficacy in metastatic melanoma and renal cell carcinoma (Dimitrijevic et al., 2012). IL-2 can also be used combined with other immunotherapeutic agents such as checkpoint inhibitors and CAR-T cell therapy to enhance their effectiveness.

#### 5.2.2 Innate immune modulators

A protein called STING is found in the endoplasmic reticulum that contributes significantly to innate immune responses. It serves as a sensor for self and pathogen-derived DNA, initiating the transcription of IFN-I genes and facilitating antigen cross presentation (Rivera Vargas et al., 2017; Donnelly et al., 2021). STING regulates protein synthesis and IFN expression through various modifications, including phosphorylation, ubiquitination, and dimerization (Corrales et al., 2017). Activation of STING in APCs by tumor cell proliferation triggers T cell-mediated adaptive immune responses, resulting in antitumor effects (Ma and Damania, 2016). In HCC, STING agonists can induce the activation and maturation of DCs and reprogram immunosuppressive macrophages into immune-activating subtypes, thereby increasing CD8^+^ T cells in the TME (Jing et al., 2019; Chen C. et al., 2021). STING also increases the expression of chemokines, such as CXCL9 and CXCL10, which attract T lymphocytes to tumor tissue, promote tumor cell killing, and initiate adaptive immune responses (Chen C. et al., 2021).

However, the IFN-β produced by the STING-TBK1-IRF3 signaling pathway can stimulate the production of immune checkpoint molecules, including PD-L1 and CTLA-4, which inhibit T-cell activation and lead to immune evasion (Garcia-Diaz et al., 2017; Morimoto et al., 2018). Therefore, the combination of STING agonists and ICI has been explored to inhibit HCC progression. This combination reduces local immunosuppression, reverses adaptive resistance, and induces systemic antitumor responses (Moore et al., 2016).

STING agonist drugs are currently being developed as a strategy to enhance cancer immunotherapy. Most of these drugs require intra-tumor injection for administration, which may have limitations in certain tumors, especially metastatic ones. However, a small molecule oral STING agonist called MSA-2 has been developed and shown to have durable antitumor immunity in mouse tumor models (Pan et al., 2020). MSA-2 stimulates the secretion of interferon β in tumors, induces tumor regression, and synergizes with anti-PD-1 treatment.

Aside from the cGAS-STING DNA sensor pathway, other pattern-recognition receptors, such as TLRs, can also recognize endogenous stress signals and strongly stimulate the immune system (Shekarian et al., 2017; Ochoa de Olza et al., 2020). TLRs are expressed diverse liver cells, including Kupffer cells, DCs, stellate cells, endothelial cells, and hepatocytes. They play a crucial role in recognizing different pathogen-associated molecular patterns.

CD47 is a glycoprotein that is frequently overexpressed in solid tumors, including HCC. This serves as a ligand for signal-regulated protein alpha (SIRPα), which is present in macrophages and DCs (Willingham et al., 2012). The interaction between CD47 and SIRPα initiates a signaling cascade that inhibits phagocytosis, enabling cancer cells to evade immune clearance. Blocking the CD47-SIRPα pathway enhances phagocyte function, promotes antigen cross presentation, and recruits T cells to the TME, leading to an adaptive antitumor immune response (Willingham et al., 2012; Autio et al., 2022; Chen H. et al., 2022). Furthermore, blocking CD47 has been observed to enhance DNA sensing specifically in DCs, but not macrophages (Xu et al., 2017). In liver tumor models, blocking the CD47-SIRPα interaction stimulates the CD103 DC-NK cell axis, resulting in increased antitumor efficacy by activating NK-cell recruitment and upregulating cytokines, such as CXCL9 and IL-12, which promote immune cell infiltration (Wang S. et al., 2022). Recent studies have focused on the development of novel recombinant SIRPα-Fc fusion protein IMM01, which simultaneously blocks the “do not eat me" signaling and activates the “eat me" signaling. This dual action exhibits antitumor activity and has the potential to convert “cold" tumors into “hot" tumors (Yu et al., 2022). Additionally, bispecific antibodies co-conjugated with Glypican-3 (GPC3) and CD47 have shown promise in enhancing the innate immune response, indicating their potential for improving HCC treatment (Du et al., 2021). Nevertheless, the effectiveness of combining CD47 blockers with other ICIs for HCC treatment requires further investigation.

#### 5.2.3 Co-stimulants

A number of studies have been conducted to investigate the use of agonists targeting co-stimulatory immune checkpoint receptors to improve the antitumor immune response. These receptors, including CD40, OX40, CD27, GITR, and ICOS, belong to the tumor necrosis factor receptor superfamily (TNFRSF) (Liu et al., 2022). They are primarily expressed on T cells, B cells, and/or NK cells, and their activation promotes signaling pathways that enhance the survival, proliferation, and effector functions of these cells (Dostert et al., 2019).

CD40, also known as TNFRSF5, is a transmembrane receptor expressed on APCs such as DCs, macrophages, and B cells. Its ligand, CD40L (CD154), is expressed on activated T cells and B cells (Diaz et al., 2021). CD40^−^CD40L co-stimulation is crucial for B-cell activation, differentiation, and memory generation in thymus-dependent humoral immune responses (Karnell et al., 2019). Binding of CD40L to DCs and macrophages stimulates the secretion of IL-12, which is essential for T-cell activation. In an *in situ* tumor model of HCC, CD40L has been shown to induce DC proliferation and maturation, resulting in slower tumor growth in mice (Gonzalez-Carmona et al., 2008; Kawashita et al., 2014b). CD40 agonists combined with anti-PD-1 have demonstrated enhanced DC maturation, increased tumor T-cell infiltration, and good antitumor efficacy (Ma et al., 2019; Diggs et al., 2021).

OX40, also known as TNFRSF4 or CD134, is primarily expressed on activated CD4^+^ and CD8^+^ T cells and NK cells. Its ligand, OX40L, can be induced on activated DCs and macrophages upon inflammatory cytokine stimulation or activation of B-cell receptors, TLRs, or CD40 (Lu, 2021). Interaction between OX40 and OX40L rapidly increases antigen presentation capacity and provides a strong co-stimulatory signal for T cells (Fu et al., 2020). Combination therapy using OX40 agonists and TLR9 agonists for HCC has shown promising results, promoting the activation of CD8^+^ and CD4^+^ T cells, suppressing regulatory T cells (Tregs) and myeloid-derived suppressor cells, and inducing immune memory responses (Zhou et al., 2021).

Overall, agonists targeting co-stimulatory immune checkpoint receptors such as CD40 and OX40 have demonstrated potential for enhancing the antitumor immune response in HCC, either alone or together with other immunotherapeutic agents. It is necessary to conduct further research to optimize their effectiveness and determine their clinical applicability.

### 5.3 Increased T-cell recruitment and infiltration

The classification of tumors as “cold" or “hot" is based on the level of T-cell infiltration within the TME. “Hot" tumors, also known as immune-inflamed tumors, have a significant presence of T cells and show immune activation. Additionally, “cold" tumors are characterized by a lack of inflammation and a scarcity of T cells, often restricted to the tumor margins (Gajewski, 2015). The influx of T cells into the TME is influenced by various processes. These include the release of tumor antigens, the uptake and presentation of these antigens by APCs, the interaction between APCs and T cells leading to T-cell activation and initiation, and the migration of T cells into the tumor (Chen and Mellman, 2013). Generation of T cells and their ability to overcome physical barriers to reach the tumor are crucial for effective antitumor immunity (Joyce and Fearon, 2015). Strategies that enhance T-cell recruitment and infiltration aim to transform “cold" tumors into “hot" tumors, thereby increasing the clinical benefits of immunotherapy.

It is worth noting that specific strategies to enhance T-cell recruitment and infiltration vary and can include various approaches, such as immune checkpoint blockade, cytokine therapy, vaccination, adoptive cell transfer, and combination therapies. Each strategy aims to promote T-cell activation, proliferation, migration, and survival within the TME, ultimately improving the antitumor immune response.

#### 5.3.1 Anti-angiogenic therapy

Tumor growth involves various processes that affect the immune system and the vascular system. These processes include the reduction of immune cell activity, the development of abnormal tumor blood vessels, and the establishment of the TME (Lopes-Coelho et al., 2021). The tumor relies on blood vessels to supply it with oxygen and nutrients for its growth (Apte et al., 2019; Anderson and Simon, 2020). However, in rapidly progressing tumors, the oxygen supply within the tumor becomes limited, leading to hypoxia. Vascular endothelial growth factor (VEGF) is released by hypoxic cancer cells and vascular endothelial cells. It plays a critical role in promoting tumor growth, invasion, and metastasis by inducing the formation of new blood vessels, a process known as neovascularization (Fukumura et al., 2018).

VEGF is a main driver of tumor angiogenesis and has immunosuppressive effects (Konishi et al., 2021). It hinders the maturation and antigen presentation of DCs, interrupting the potency of T cells against the tumor (Gabrilovich et al., 1996). Furthermore, VEGF stimulates immunosuppressive cells to mobilize and proliferate, such as tumor-associated macrophages (TAMs), Tregs, and myeloid-derived suppressor cells (MDSCs) (Fukumura et al., 2018). These cells further release VEGF and other immunosuppressive cytokines, inhibiting the proliferation and activation of naive CD8^+^ T cells and suppressing immune responses. The tumor vasculature selectively recruits immunosuppressive immune cells into the TME, contributing to immune escape. Anti-angiogenic drugs can increase the infiltration of immune cells and make immunotherapy more effective. They promote antigen presentation, activate the tumor immune response, reverse VEGF-induced immunosuppression, promote the migration and infiltration of immune lymphocytes, enhance the activity of T lymphocytes and immune effector molecules, normalize the tumor vascular system, and improve therapeutic drug delivery (Lee WS. et al., 2020; Feng et al., 2020; Kudo, 2020).

In HCC, anti-angiogenic therapies commonly involve large-molecule monoclonal antibodies targeting VEGF or vascular endothelial growth factor receptor (VEGFR), as well as small molecule tyrosine kinase inhibitors (TKIs) targeting multiple receptors. Examples of these therapies include bevacizumab, ramucirumab, sorafenib, and Lenvatinib (Bruix et al., 2021). These drugs inhibit VEGF/VEGFR or multiple signaling pathways, exerting antitumor effects and improving outcomes in advanced liver cancer (Cheng et al., 2009). Notably, there is evidence that lenvatinib enhances the efficacy of anti-PD-1 treatment by inhibiting the FGFR4-glycogen synthase kinase 3β axis, promoting T-cell killing of HCC cells, and inhibiting Treg differentiation (Kudo et al., 2018). This combination approach improves the response to anti-PD-1 treatment. Overall, targeting angiogenesis and the tumor vasculature through anti-angiogenic therapies can increase the immune response against cancer and improve the effectiveness of immunotherapy in HCC.

#### 5.3.2 T-cell transport regulators

In immune-altered and excluded tumors, there is a lack of T-cell infiltration within the tumor bed, with T cells primarily accumulating around the tumor margin. This absence of T-cell recruitment signals has been related to poor response to immunotherapy in HCC and other tumors. Chemokines play a crucial role in influencing the trafficking of effector T lymphocytes to the tumor site. Chemokines, such as CXCL9, CXCL10, CX3CL1, CXCL16, CCL2, and CCL5, are involved in T-cell recruitment (van der Woude et al., 2017; Maimela et al., 2019). The deficiency of these chemokines, particularly the TH1-type chemokines CXCL9 and CXCL10, can result in T-cell exclusion. This phenomenon has been observed in HCC.

MCT4, an overexpressed lactate transporter protein in HCC, has been successfully targeted in a mouse model (Fang et al., 2023). Inhibition of MCT4 led to an increased expression of CXCL9 and CXCL10, enhanced recruitment and activity of CD8^+^ T cells, and improved patient prognosis. The lack of chemokines in tumors may be attributed to epigenetic regulation. DNA methylation mediated by DNA methyltransferase (DNMT) can result in the deletion of chemokine expression, such as CCL5, resulting in a deficiency in CD8^+^ T-cell infiltration (McGrail et al., 2018). DNA demethylating agents combined with histone deacetylase inhibitors have shown the ability to enhance chemokine levels, promote T-cell infiltration, and reverse immune resistance in certain tumor models (Topper et al., 2017). However, in HCC, overexpression of CCL5 may recruit Tregs via p38-MAPK signaling, leading to immune escape (Sun et al., 2021). Selective HDAC8 inhibitors have been found to alter the epigenetic landscape of HCC cells and induce the production of T-cell recruitment chemokines in preclinical models, thereby increasing CD8^+^ T-cell infiltration. Some chemokines, such as CXCL12 and CXCL8, are related to a decrease in T-cell presence within tumors (Mortezaee, 2020).

In a word, targeting the epigenetic regulation of TH1-derived chemokines in combination with immunotherapy holds promise for the treatment of HCC and other immune-excluded tumors. By enhancing T-cell recruitment and overcoming immune exclusion, this approach aims to improve the effectiveness of immunotherapy.

### 5.4 Enhanced recognition and cytotoxic activity of effector immune cells

#### 5.4.1 Adoptive cell transfer therapy

Adoptive cell transfer therapy (ACT) involves the retrieval of immune cells from a patient, followed by their cultivation and processing to enhance their targeted killing capacity and quantity. The treated cells are then reintroduced into the patient’s body to combat cancer cells (Zhang et al., 2019). Chimeric antigen receptor T-cell (CAR-T) therapy is recognized as a potentially curative approach for cancer treatment. CAR-T cells, distinct from TIL therapy and engineered TCR therapy, possess the capacity to independently identify tumor cells, bypassing the requirement for MHC-I antigen presentation. Their activation is solely triggered by binding to specific target antigens, resulting in precise eradication of tumor cells (Larson and Maus, 2021). In 2017, the FDA approved two CAR-T cell immunotherapies for the treatment of relapsed or refractory diffuse large B-cell lymphoma (DLBCL) and acute lymphoblastic leukemia (ALL) targeting CD19 (Chen et al., 2023). Since then, extensive research has been conducted on various hematological and solid tumors. However, CAR-T cell therapy encounters challenges when treating solid tumors, primarily attributed to the identification of target antigens for T-cell engineering. The majority of antigens in solid tumors are shared with normal tissues, posing a significant risk of off-tumor toxicity (Rosenberg and Restifo, 2015; Flugel et al., 2023). Nevertheless, CAR-T cell therapy, along with TCR therapies, holds potential to enhance the efficacy in “cold" tumors. ACT therapy amplifies T-cell numbers, enhances tumor specificity, and endows T cells with a novel targeted activation function. Furthermore, combining ACT therapy with anti-PD-1 has demonstrated improved anticancer effects against solid tumors (Wiede et al., 2022). GPC3, an oncoprotein implicated in HCC progression, serves as a potential immunotherapeutic target for HCC. There is a high level of GPC3 expression in various solid tumors, while it is absent in healthy adult tissue (Szoor et al., 2017). Clinical trials of HCC have shown that GPC3-CAR-T cells inhibit tumor growth (NCT03198546, NCT02395250, and NCT03146234) (Shi et al., 2020). Additionally, GPC-3-specific CAR-T cells expressing IL3 and IL15, along with the secretion of IL-7 and CCL19, have exhibited remarkable expansion and robust antitumor responses in HCC (Batra et al., 2020; Pang et al., 2021). Furthermore, genetic engineering utilizing allogeneic cells and other immune cell subsets, in combination with CAR technology, has been employed. For instance, amplified Vδ15 T cells modified with GPC-3 CAR and sIL-3 have demonstrated potent cytotoxic activity against HCC (Makkouk et al., 2021). Nevertheless, it is essential to acknowledge that ACT therapy for HCC presents potential risks of off-target toxicity and resistance, particularly in conjunction with ICIs. Hence, optimizing patient safety by controlling toxicity represents a crucial aspect for future advancements in this field.

#### 5.4.2 Therapeutic cancer vaccines

Vaccine-based cancer therapy holds significant promise in enhancing the recognition of immune cells targeting cancer. However, selecting the most suitable antigen for therapeutic cancer vaccines presents a major challenge. The ideal antigen must exhibit specific expression on tumor cells and possess high immunogenicity (Saxena et al., 2021). In recent years, the emergence of neoantigen vaccines has revolutionized this field. Neoantigens, also known as tumor-specific antigens (TSAs), are abnormal antigens that result from mutations in tumor cells and are exclusively expressed on these cells, enabling immune recognition. Notably, the NeoVax vaccine demonstrated a robust and durable antitumor response in melanoma patients, persisting for up to 4 years (Hu Z. et al., 2021).

Despite the potential benefits of cancer vaccines, the treatment of HCC has faced challenges, possibly due to the specific tumor immune microenvironment or obstacles such as immunosuppressive mechanisms and cellular dysfunction. However, several studies have confirmed the synergistic effect of combining ICIs with cancer vaccines in HCC (Chen et al., 2020; Silva et al., 2020; Shi et al., 2021; Teng et al., 2021; Yarchoan et al., 2021; Bai et al., 2022). HCC vaccination strategies can be categorized into DC-based or peptide-based vaccines. Notably, neoantigen-based DC vaccines (neo-DCs) have shown tremendous potential due to their minimal off-target effects, high specificity, and strong immunogenicity. For instance, Wang et al. developed an acidic/photosensitive DC-based neoantigen nanovaccine that enhances the anticancer immune response and converts “cold" tumors into “hot" tumors, thus improving the efficacy of immunotherapy for HCC (Wang Y. et al., 2022).

In two additional studies, DC vaccines were combined with anti-PD-1 monoclonal antibodies in HCC, leading to improved cytokine secretion, activation, and proliferation of CD8 T cells. This combination approach resulted in enhanced tumor regression and prolonged overall survival in mice and demonstrated promising results (Shi et al., 2021; Teng et al., 2021). The primary antigens utilized in peptide-based vaccines for HCC include GPC3, delta-catenin, and methemoglobin. For example, Chen K. et al. developed the XCL1-GPC3 fusion gene peptide protein for a mouse model of HCC with a hepatitis B background. The XCL1-GPC3 vaccine increased the infiltration of tumor-specific cytotoxic T cells into the tumor bed and improved the response of HCC to ICI despite the presence of a suppressive microenvironment (Chen et al., 2020). Another vaccine construct based on ASPH and λ-phage demonstrated a potent synergistic antitumor immune response in preclinical models when combined with ICI (Bai et al., 2022). It is evident from these findings that there is significant potential for combining ICIs with vaccines to enhance therapeutic outcomes in HCC.

### 5.5 Other factors associated with immunotherapy efficacy

Improving cancer immunotherapy’s effectiveness by combining it with standard clinical treatments and modulating the host’s own system through dietary and exercise interventions, as well as the regulation of the gut microbiota, has gained significant attention in recent years (Li X. et al., 2022). While the liver does not possess its own microbiome, it is connected to the intestine through the portal vein, forming the intestine–hepatic axis (Weng et al., 2019). The gut microbiota, a complex ecosystem, plays a crucial role in gastrointestinal, liver, and pancreatic diseases. Its impact on the activation of innate and adaptive immunity is particularly relevant to the success of cancer immunotherapy. Dysbiosis of the gut microbiota can lead to chronic inflammation, tissue fibrosis, and disturbances in lipid metabolism, thereby increasing the risk of HCC (Yu and Schwabe, 2017). Preclinical models have validated the mechanistic relationship between the gut microbiota and antitumor immunity, with germ-free mice simulating patient-derived microbial communities (Routy et al., 2018). Intestinal microbial patterns or pathogens can cross the mucosal barrier and reach tumors via the bloodstream, eliciting a robust immune response. Microbiota and their metabolites, such as short-chain fatty acids (SCFAs), influence systemic immunity by promoting the development and differentiation of T cells and regulating the TME (Aghamajidi and Maleki Vareki, 2022). How systemic therapy affects the gut microbiota, including ICIs, has been shown to affect the sensitivity of HCC cells to apoptosis induction and the response of patients to ICIs (Dzutsev et al., 2017). Inter-individual variations in the gut microbiota may contribute to the heterogeneous response to ICI therapy among patients. Unlike host genetics, the gut microbiota can be modified through approaches such as fecal microbiota transplantation (FMT), probiotics, prebiotics, and antibiotics. FMT has been used to treat gastrointestinal disorders and has been explored combining ICI immunotherapy in preclinical and clinical studies. In conclusion, a healthy diet and lifestyle can influence cancer development by modifying the composition of the gut microbiota and inducing local and systemic immune responses.

## 6 Clinical trials of ICIs in HCC

**FIGURE 6:**
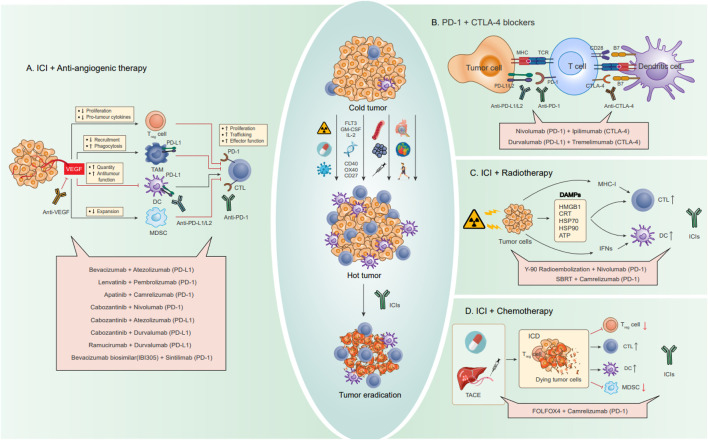
Combination strategies for HCC to enhance the effect of ICI blocking therapy, including drug combinations. Through some strategies summarized in the previous paragraph, cold tumors are transformed into hot tumors and then combined with ICIs to eventually eradicate tumors. **(A)** Combination therapy with anti-angiogenic drugs. The angiogenic factor VEGF can directly regulate various immune cells, causing immunosuppression. Anti-VEGF can promote antigen presentation and migration and infiltration of immune lymphocytes, improve the activity of T lymphocytes and immune effector molecules, and reverse VEGF-induced immunosuppression. **(B)** Combination therapy of PD-1 and CTLA-4 dual ICIs. Activation of PD-1/PD-L1 and CTLA-4 can be blocked by anti-PD-1/PD-L1 and CTLA-4 antibodies, respectively. The combined use of PD-1 inhibitors and CTLA-4 inhibitors creates a synergistic effect. **(C)** Combination therapy with radiotherapy. By inducing ICDs in tumor cells, radiotherapy can trigger an immune response while releasing specific signaling molecules, DAMPs, including HMGB1, CRT, HSP70, HSP90, and ATP. In addition, radiotherapy also promotes CTL and DC by upregulating MHC-I and IFNs. **(D)** Combination therapy with chemotherapy. Conventional chemotherapy drugs and interventional arterial chemotherapy can cause ICD by inducing DNA damage, promote the infiltration of inflammatory APCs into tumors, and induce and activate tumor-specific CD8^+^ T cells, while reducing the activity of suppressor immune cells (T_reg_ cells and MDSC).

Four configurations stemming from the amalgamated strategies delineated earlier have progressed into clinical investigations, yielding certain outcomes (Figure 6). Among these, the pairing of ICIs with anti-angiogenic therapy stands as the most extensively scrutinized and has demonstrated the most favorable outcomes in clinical scenarios. Furthermore, the integration of ICIs with oncolytic viruses has ventured into clinical experimentation, although the extent of research in this avenue remains limited. Based on the search criteria and screening, a total of 92 studies related to HCC and ICIs were obtained from the ClinicalTrials.gov database. Among them, a total of 23 studies have been completed, while 69 studies are currently ongoing. The main combination therapies applied to HCC are summarized in Table 2 and Figure 6.

**TABLE 2:** Summary of clinical trials of targeted ICIs in combination with HCC.

Drug	Trial identifier	Target	Phase	N	Design	ORR %	DCR %	PFS months	OS months
Nivolumab+ipilimumab	NCT01658878 (CheckMate040)	PD-1+CTLA-4	I/II	148	Arm A: nivolumab 1 mg/kg + ipilimumab 3 mg/kg iv q3w (4 doses) and then nivolumab 240 mg iv q2w	32	49	NA	22.8
Durvalumab+tremelimumab	NCT02519348	PD-L1+CTLA-4	I/II	332	T300 + D: tremelimumab 300 mg + durvalumab 1,500 mg (1 doses) and then durvalumab 1,500 mg iv q4w	24	NR	NA	18.7
Durvalumab+tremelimumab	NCT03298451	PD-L1+CTLA-4	III	1,171	STRIDE: tremelimumab 300 mg (1 dose) plus durvalumab 1,500 mg iv q4w	20.1	NA	NA	16.43
Atezolizumab+bevacizumab	NCT03434379 (IMbrave150)	PD-L1+ VEGF	III	501	Atezolizumab 1200 mg + bevacizumab 15 mg/kg iv q3w	33	NA	6.9	19.2
Tiragolumab+atezolizumab+bevacizumab	NCT04524871	TIGIT + PD-L1+ VEGF	Ib/II	58	Tiragolumab 600 mg + atezolizumab 1200 mg + bevacizumab 15 mg/kg iv q3w	42.5	NA	11.1	NA
Pembrolizumab + lenvatinib	NCT03006926 (KEYNOTE-524)	PD-1+ VEGF	I	104	Pembrolizumab 200 mg iv (1 doses) + lenvatinib 8/12 mg po qd	36–46	NA	8.6–9.3	22
Camrelizumab+apatinib	NCT03006926	PD-1+ VEGF	I	18	Camrelizumab 200 mg iv q2w + apatinib 125-500 mg po qd	50	93.8	5.8	NR
Nivolumab+cabozantinib	NCT01658878 (CheckMate040)	PD-1+ VEGF	I/II	71	Nivolumab 240 mg iv q2w + cabozantinib 40 mg po qd	17	NA	5.1	20.2
Nivolumab+cabozantinib + ipilimumab	NCT01658878 (CheckMate040)	PD-1+ VEGF + CTLA-4	I/II	71	Nivolumab 3 mg/kg iv q2w + cabozantinib 40 mg po qd + ipilimumab 1 mg/kg iv q6w	29	NA	4.3	22.1
Camrelizumab+apatinib	NCT03463876(RESCUE)	PD-1+ VEGF	II	70/120	Camrelizumab 200 mg (≥50 kg)/3 mg/kg (<50 kg) iv q2w + apatinib 250 mg po qd	34.3/22.5	NA	5.7/5.5	NR
Camrelizumab+apatinib	NCT02942329	PD-1+ VEGF	I/II	60	Camrelizumab 200 mg (3 mg/kg for underweight patients) iv q2w + apatinib 250 mg po qd	30.8	NA	NA	NA
Camrelizumab+apatinib	NCT03092895	PD-1+ VEGF	I/II	157	Camrelizumab 3 mg/kg iv q2w + apatinib 125,250,375, or 500 mg po qd	10.7	NA	3.7	13.2
Atezolizumab + cabozantinib	NCT03755791(COSMIC-312)	PD-L1+ VEGF	III	432	Atezolizumab 1,200 mg iv q3w + cabozantinib 40 mg po qd	NA	NA	6.8	15.4
Durvalumab + cabozantinib	NCT03539822	PD-L1+ VEGF	I	35	Durvalumab 1,500 mg iv + cabozantinib 40 mg po qd	30	83.3	4.5	8.7
Sintilimab + bevacizumab biosimilar (IBI305)	NCT03794440 (ORIENT-32)	PD-1+ VEGF	II- III	380	Sintilimab 200 mg iv q3w + IBI305 15 mg/kg iv q3w	NA	NA	4.6	NR
Durvalumab + ramucirumab	NCT02572687	PD-L1+ VEGF	I	85	Ramucirumab 8 mg/kg iv q2w + durvalumab 750 mg iv q2w	11	NA	4.4	10.7
Sintilimab + apatinib + capecitabine	NCT04411706	PD-1+ VEGF+Chemotherapy	II	46	Sintilimab 200 mg iv q3w + apatinib 250 mg po qd + capecitabine 1,000 mg/m^2^ po bid	50	91.3	9	NR
Camrelizumab + FOLFOX4	NCT03092895	PD-1+ Chemotherapy	I/II	157	Camrelizumab 3 mg/kg iv q2w + FOLFOX4 q2w	29.4	79.4	7.4	11.7
Spartalizumab + FGF401	NCT02325739	PD-1+ FGFR4	I/II	172	Spartalizumab 300 mg iv q3w + FGF401 80 mg po qd	16.7	50	NA	NA
Nivolumab + mogamulizumab	NCT02476123	PD-1+ CCR4	I	118	Nivolumab 3 mg/kg iv q2w + mogamulizumab 0.3 or 1.0 mg/kg iv q1w	NA	NA	NA	NA
Nivolumab + Y-90 radioembolization	NCT03033446	PD-1+ radiotherapy	II	40	Y-90 radioembolization + nivolumab 240 mg iv q2w	30.6	NA	NA	NA
Camrelizumab+SBRT	NCT04193696	PD-1+ radiotherapy	II	39	SBRT Dt-PGTV = 40Gy/10fractions,Dt-PGTV = 30Gy/10fractions,Dt-PGTV = 20Gy/10fractions + camrelizumab 200 mg iv q3w	52.4	NA	5.8	14.2
Tremelimumab + RFA/TACE	NCT01853618	CTLA-4+ RFA/TACE	I/II	61	Tremelimumab 3.5/10 mg/kg iv 6 doses q4w + RFA/TACE on day 36	NA	NA	7.4	12.3
Avelumab + TACE + SBRT	NCT03817736(START-FIT)	PD-L1+ TACE + SBRT	II	33	TACE on day 1+ SBRT (27·5–40·0 Gy in five fractions) on day 28+avelumab 10 mg/kg iv q2w	NA	NA	NA	NA
Nivolumab + Pexa-Vec	NCT03071094	PD-1+ oncolytic	I/II	14	Pexa-Vec 3 bi-weekly intra-tumoral (IT) injections of 10^9 pfu at day 1 and weeks 2 and 4 + nivolumab iv q2w	33.3	NA	NA	NA

NA: not applicable; NR: not reached.

Among the most effective and extensively studied combination therapies for HCC involves combining ICIs with anti-angiogenic agents or multi-target tyrosine kinase inhibitors. For example, the combination of atezolizumab (anti-PD-L1) and bevacizumab (anti-VEGF antibody) was approved by the FDA in May 2020 to treat non-hepatocellular carcinoma. In a phase III study, the atezobev group showed an ORR of 33% and a median OS of 19.2 months, compared to an ORR of 13% and a median OS of 13.4 months in the sorafenib group. The combination demonstrated good efficacy with manageable adverse events (Cheng et al., 2022). In addition, at the latest ASCO Annual Meeting 2023, the MORPHEUS-liver study offers a new combination. This phase Ib/II trial (NCT04524871) assessed the efficacy of tiragolumab (tira) combined with atezo and bev in advanced HCC. Tira is a novel cancer immunotherapy that targets TIGIT. This trial’s results showed that the ORRs of the tira + atezo + bev arm and the control group (atezo + bev) were 42.5% and 11.1%, respectively. The median PFS is 11.1 months and 4.2 months, respectively. The addition of tira to atezo + bev produces encouraging efficacy and safety compared to atezo + bev. According to these findings, tira + atezo + bev may be a promising treatment option for patients with advanced HCC as a first-line treatment.

Another combination therapy being explored is lenvatinib (tyrosine kinase inhibitor) in combination with pembrolizumab (anti-PD-1) in an open-label, multicenter trial (NCT03006926) (Cheng et al., 2022). The study showed an ORR of 36%–46%, median duration of response (DOR) of 8.6–12.6 months, mPFS of 8.6–9.3 months, and mOS of 22 months. These results were superior to those from a phase III trial (NCT02702401), indicating improved antitumor activity with the combination therapy.

Camrelizumab (anti-PD-1) and apatinib (anti-VEGFR-2) combination therapy has also been studied in advanced HCC (Xu et al., 2021). The ORR for first-line and second-line patients was 34.3% and 22.5%, respectively, with mid-position PFS of 5.7 and 5.5 months. A phase III trial (NCT03764293) evaluating the combination of camrelizumab and apatinib in first-line HCC treatment is currently ongoing.

Cabozantinib in combination with atezolizumab (anti-PD-L1) has been studied in a randomized phase III trial (NCT03755791) comparing it with sorafenib in patients without prior systemic therapy for advanced HCC (Kelley et al., 2022). In the combination group, the median PFS was 6.8 months, and in the sorafenib group, it was 4.2 months, while the median OS was 15.4 and 15.5 months, respectively.

The combination of sintilimab (anti-PD-1) and bevacizumab biosimilar (IBI305) versus sorafenib is being investigated in an open-label, randomized phase II-III trial called ORIENT-32 (NCT03794440). Preliminary results showed a median PFS of 4.6 months in the sintilimab–bevacizumab biosimilar group compared to 2.8 months in the sorafenib group.

The combination of ICIs and angiogenesis inhibitors is also being studied in several trials for HCC, such as durvalumab (anti-PD-L1) plus bevacizumab (NCT03847428) and CS1003 (anti-PD-1) plus lenvatinibin addition to the atezolizumab and bevacizumab combination, another approved combination for unresectable HCC is tremelimumab (anti-CTLA-4) combined with durvalumab (anti-PD-L1). This combination demonstrated favorable results in a multicenter, randomized phase Ⅲ trial (NCT03298451). The mOS was 16.43 months with STRIDE (tremelimumab plus durvalumab), 16.56 months with durvalumab, and 13.77 months with sorafenib. Overall survival at 36 months was 30.7%, 24.7%, and 20.2%, respectively. The trial was designed to compare STRIDE to sorafenib and found that the STRIDE and durvalumab groups had higher antitumor activity compared to sorafenib. In addition, they found that tremelimumab increased the overall survival benefit of durvalumab over time, which also reflects that STRIDE has a higher overall survival in comparison with durvalumab. Additionally, a phase II study (NCT05440864) evaluating tremelimumab and durvalumab in resectable liver cancer was recently published. Nivolumab (anti-PD-1) plus ipilimumab (anti-CTLA-4) is another treatment option for dual immune checkpoint blockade. In the CheckMate-040 (NCT01658878) study, the combination group showed an ORR of 32% and a median OS of 22.8 months, compared to an ORR of 15% and a median OS of 16.4 months in the nivolumab monotherapy group (Reig and Sanduzzi-Zamparelli, 2022; Yau et al., 2022). Although adverse events occurred more frequently in the combination group, the safety profile was consistent with nivolumab and ipilimumab monotherapy. A phase III trial, CheckMate-9DW (NCT04039607), is currently underway to compare nivolumab plus ipilimumab with standard care (sorafenib or lenvatinib) in patients with advanced HCC who have not received systemic therapy.

There are many other immune checkpoint molecules; for example, the combination of LAG-3 and TIM-3 with PD-1/PD-L1 or CTLA-4 inhibitors is also being investigated for the treatment of HCC. Clinical trials such as a phase I/II trial evaluating relatlimab (anti-LAG-3) + nivolumab (anti-PD-1) + bevacizumab (NCT05337137), a phase I trial evaluating REGN3767 (anti-LAG-3) in combination with REGN2810 (anti-PD-1) (NCT03005782), and a phase I trial evaluating LY3321367 (anti-TIM-3) and LY3300054 (anti-PD-L1) (NCT03099109) are exploring the antitumor activity and safety of these combinations in advanced solid tumors. In a phase II trial (NCT03033446), the combination of Y90 resin microsphere radioembolization and nivolumab showed promising results in advanced HCC, with a 3% complete response rate and a 28% partial response rate, resulting in an ORR of 30.6% (Tai et al., 2021). Similarly, the combination of stereotactic body radiotherapy and camrelizumab (NCT04193696) demonstrated encouraging efficacy, with an ORR of 52.4%, mPFS of 5.8 months, and mOS of 14.2 months (Li JX. et al., 2022). In recent studies, researchers have explored the triple therapy of stereotactic body radiotherapy with dual ICIs (nivolumab plus ipilimumab) and showed an ORR of 57%, mPFS of 11.6 months, and mOS of 41.6 months, indicating excellent antitumor activity (Juloori et al., 2023). Another triple therapy combining stereotactic body radiotherapy, TACE, and avelumab (NCT03817736) also showed promising results (Chiang et al., 2023). Evidence for the combination of chemotherapy and ICI was revealed in an open-label phase II trial (NCT03092895). The combination of chemotherapy (FOLFOX4 or GEMOX) with camrelizumab (SHR-1210) in advanced liver cancer demonstrated good antitumor activity, with an ORR of 29.4%, disease control rate (DCR) of 79.4%, mPFS of 7.4 months, and mOS of 11.7 months, compared to camrelizumab monotherapy (Qin et al., 2020; Li et al., 2021). OVs and ICI have also demonstrated promise when combined. In a phase I/II trial, the combination of the oncolytic virus Pexa-Vec with nivolumab in advanced HCC achieved an ORR of 33.3%, although it was associated with relatively high toxicity.

Please refer to Table 2 for a summary of the remaining trials.

## 7 Conclusion and future perspectives

Targeted combination immunotherapy has emerged as a novel first-line treatment option for HCC; however, the majority of patients derive limited benefit from ICIs. Recent research has indicated that HCC tumors predominantly belong to the “cold" tumor category, resulting in inadequate response to ICIs. To address this critical issue, we must approach it from three key aspects in order to uncover effective solutions for unleashing the efficacy of ICIs in HCC treatment.

First, identifying predictive biomarkers plays a crucial role in aiding clinicians in patient selection and personalized treatment decisions. Biomarkers such as PD-L1 expression, TMB, and mismatch repair/microsatellite instability (MMR/MSI) have been used to assess the efficacy of ICIs (Doroshow et al., 2021). Patients with high PD-L1 expression and TMB are more likely to benefit from immunotherapy (Rizvi et al., 2018). Although PD-L1 has gained FDA approval as a predictive biomarker in certain cancers, its significance in HCC remains inconclusive. Furthermore, the potential role of the gut microbiota as a biomarker requires further investigation. Recent advancements in single-cell sequencing techniques and spatial transcriptome sequencing offer valuable insights into tumor immunophenotypes and immune infiltration patterns (Feng et al., 2023), paving the way for potential clinical treatment options.

Second, in-depth exploration of factors contributing to “cold" tumors, such as enhanced antigen presentation, increased T-cell recruitment and infiltration, and improved recognition of effector immune cells, will facilitate the optimization of strategies to enhance the effectiveness of ICIs in HCC.

Third, combining immunotherapeutic approaches with modalities such as radiotherapy, chemotherapy, oncolytic viruses, and cancer vaccines can activate “cold" tumors and enhance the response to ICI treatment, potentially improving overall survival. Overcoming challenges such as tumor heterogeneity, individualized immunotherapy, striking a balance between efficacy and toxicity, and translating preclinical findings into clinical outcomes necessitate further investigation.

In summary, translational research is crucial for enhancing the efficacy of ICI treatment in HCC, with advancements in biotechnology and artificial intelligence (AI) offering promising prospects for identifying and treating “hot" tumors in the future.
